# Cell Type- and Phase-Dependent Immune Checkpoint Targeting in Sepsis: TIM-3 as a Model for Precision Immunomodulation

**DOI:** 10.3390/pharmaceutics18101343

**Published:** 2026-10-21

**Authors:** Jialiu Luo, Peidong Zhang, Shuaipeng Gu, Zhiqiang Lin, Yuhao Wu, Zhao-Hui Tang

**Affiliations:** Department of Trauma Surgery, Emergency Surgery & Surgical Critical, Tongji Trauma Center, Tongji Hospital, Tongji Medical College, Huazhong University of Science and Technology, Wuhan 430030, China

**Keywords:** sepsis, immune checkpoints, TIM-3, immune endotypes, precision immunomodulation

## Abstract

Sepsis is a dysregulated host response in which hyperinflammation, immunoparalysis, lymphoid depletion, myeloid dysfunction, tissue injury and defective pathogen control frequently coexist and evolve over time. Immune checkpoint pathways have therefore attracted interest as candidates for immune restoration, but receptor upregulation alone is an unreliable therapeutic signal. This review develops a cell type- and phase-dependent framework for immune checkpoint targeting in sepsis, using TIM-3 as a model checkpoint node. We first discuss the conceptual pitfalls in translating checkpoint blockade from cancer and chronic infection to acute heterogeneous sepsis. We then examine why the immune phase should be defined by dynamic host response states rather than by the clock time and why checkpoint signals must be interpreted according to their cellular source, including effector lymphocytes, NKT cells, CD4+ T cells, monocytes/macrophages, dendritic cells and suppressive myeloid populations. TIM-3 illustrates this complexity. In sepsis-relevant studies, TIM-3 has been linked to NKT cell and hepatic CD8+ T-cell apoptosis, CD4/HMGB1/TIM-3-associated hyporesponsiveness, Galectin-9-related ligand effects and potential myeloid or tissue-resident functions involving phosphatidylserine-positive debris, extracellular vesicles and tissue homeostasis. These observations argue against treating TIM-3 as a single-purpose inhibitory receptor or assuming that antibody blockade, ligand administration, receptor mutation and Galectin–glycan interference perturb the same biology. We propose that future checkpoint-directed studies should enroll immune states rather than syndromic sepsis alone; integrate the inflammatory burden, innate immune competence, lymphoid function and ligand context; and prioritize mechanistic endpoints such as lymphocyte apoptosis, IFN-γ recovery, monocyte HLA-DR restoration, LPS-induced TNF responses, bacterial clearance and secondary infection. Future precision immunomodulation studies in sepsis should evaluate defined checkpoint-associated circuits rather than infer therapeutic relevance from receptor expression alone.

## 1. Introduction

Sepsis is no longer viewed simply as an overwhelming inflammatory reaction to infection [1]. The Sepsis-3 definition frames it as life-threatening organ dysfunction caused by a dysregulated host response, and this wording remains crucial [2]. Recent international guidance has sharpened the clinical side of this problem: early recognition, antimicrobial stewardship, source control and organ support remain the backbone of care, while sepsis continues to carry a large global burden and long-term physical, cognitive and psychological consequences for survivors [3]. What is still missing is not a broad supportive measure but a way to treat the immune disorder itself without exacerbating the wrong part of the host response.

This has proven difficult, because sepsis is not one immune disease [4]. A patient may arrive with cytokine-driven inflammation, lymphocyte loss, monocyte deactivation, endothelial injury, metabolic stress and incomplete pathogen control combined. The old “early storm, late paralysis” model helped to move the field beyond pure anti-inflammatory thinking, but it is insufficient for real patients. Current precision immunotherapy frameworks emphasize that hyperinflammation and immunoparalysis can coexist, arise from different molecular drivers and change within the same patient over time [5,6]. Recent precision medicine reviews further argue that immune profiling should move from descriptive biomarkers toward clinically actionable enrichment strategies. This translational view also reframes sepsis immune dysfunction as the disruption of host resistance, disease tolerance, resilience and resolution, rather than as a simple shift from inflammation to paralysis [7,8].

Newer data make this point more difficult to ignore. Single-cell profiling has identified sepsis-associated immune cell states, including an expanded CD14+ monocyte state validated across public cohorts [9]. Transcriptomic studies in critically ill patients show that immune endotypes are not fixed labels; they can shift during ICU hospitalization and carry different risks for secondary infection and adverse outcomes [10]. Even pre-existing immune status matters. In immunocompromised patients with sepsis, hematologic malignancy has been strongly associated with a hyperinflammatory subphenotype, demonstrating that “immunocompromised” does not always mean “immunologically quiet” [11].

This is the background against which immune checkpoint targeting has entered the sepsis field. The logic is understandable. Septic patients may develop lymphocyte exhaustion, apoptosis, impaired antigen presentation and poor antimicrobial defense; therefore, releasing inhibitory pathways such as programmed cell death protein 1/programmed death-ligand 1 (PD-1/PD-L1), TIM-3, B- and T-lymphocyte attenuator (BTLA) or lymphocyte-activation gene 3 (LAG-3) appears attractive. It fits well with the broader movement toward precision medicine, which has now been explicitly listed among major sepsis research priorities [12]. However, the evidence demands caution. A systematic review of clinical studies found that some checkpoint molecules, particularly CD4+ T-cell PD-1 and monocyte PD-L1, are often increased in septic patients compared with healthy controls, but the findings are less consistent in comparisons with critically ill non-septic patients and are frequently confounded by the illness severity and comorbidities [13]. Accordingly, checkpoint expression alone should not be interpreted as a therapeutic indication. Even for T-cell exhaustion, the case for blockade in sepsis remains less mature than in cancer or chronic viral infection [14].

TIM-3 illustrates this problem well. Sepsis-relevant studies have linked TIM-3 to lymphoid apoptosis and adverse outcomes, while other work suggests that TIM-3 can also participate in inflammatory restraint and ligand-dependent tissue regulation [15]. This makes TIM-3 a useful model, not because it is an obvious target to block but because it exposes the central difficulty of checkpoint biology in sepsis: the same receptor may carry different meanings across lymphoid and myeloid compartments, disease phases, ligands and tissue contexts.

**Figure 1:**
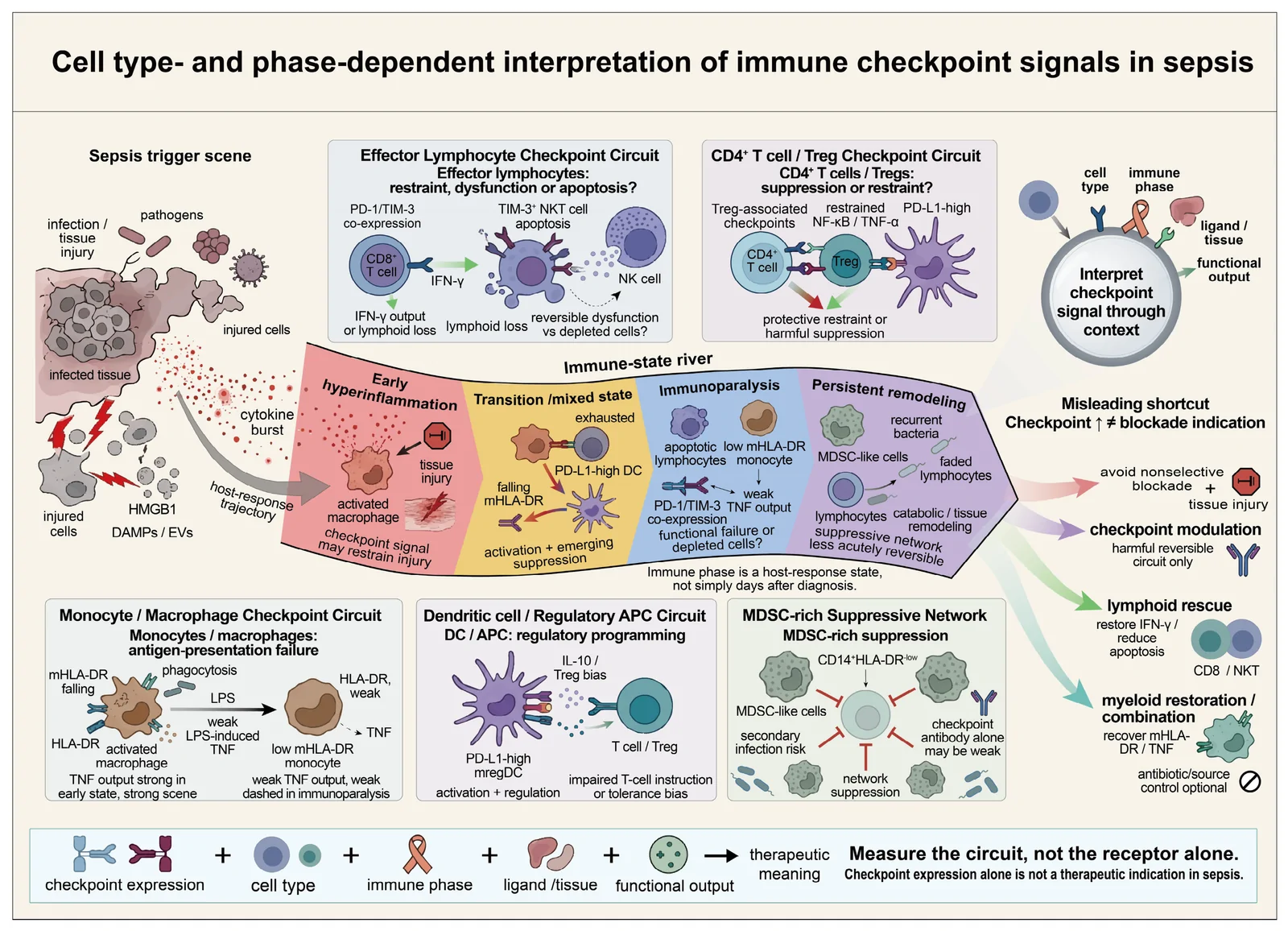
Cell type- and phase-dependent interpretation of immune checkpoint signals in sepsis. Immune checkpoint expression in sepsis should not be interpreted as a direct indication for checkpoint blockade. The same checkpoint signal may reflect tissue-protective restraint during early hyperinflammation, mixed activation and emerging suppression during transition, reversible lymphocyte dysfunction or apoptosis-prone lymphoid loss during immunoparalysis, myeloid antigen-presentation failure, regulatory dendritic cell programming, MDSC-rich suppressive remodeling or persistent immune dysfunction depending on the checkpoint-expressing cell type and immune phase. Therapeutic interpretation therefore requires the integration of the cell type, immune phase, ligand and tissue context and functional output. The goal is to measure the immune circuit rather than receptor upregulation alone. The phase labels are conceptual dominant response states rather than fixed chronological stages, and overlapping or mixed states may coexist within the same patient.

This review therefore argues that immune checkpoint targeting in sepsis should be understood through a cell type- and phase-dependent framework (Figure 1). The useful question is no longer merely “which checkpoint should we block?”. The important question is less direct but more clinically relevant: which checkpoint should be modulated, in which cell type, at which immune phase and under which ligand or tissue context? To avoid conflating mechanistic plausibility with translational evidence, we distinguish throughout this review among human interventional evidence, human observational evidence, human ex vivo functional evidence, causal evidence from experimental sepsis models and mechanistic evidence derived from non-sepsis systems. Findings from non-sepsis models are used to establish mechanistic plausibility, rather than as direct evidence of TIM-3 function in human sepsis, and therapeutic implications are weighted according to the level and disease relevance of the supporting evidence.

## 2. Literature Search and Evidence Appraisal

This work was conducted as a structured narrative and mechanistic review rather than a systematic review or meta-analysis. The relevant literature was identified through searches of PubMed and the Web of Science Core Collection from database inception to 30 August 2026. Search terms were organized around the major themes of this review and included combinations of terms related to sepsis, immune checkpoints, TIM-3/HAVCR2, immune dysfunction, immune endotypes, immunoparalysis, Galectin-9, HMGB1, CEACAM1, phosphatidylserine and checkpoint-directed immunotherapy. The reference lists of key primary studies and relevant reviews were also examined to identify additional pertinent publications. The principal search strategies and their purposes are provided in Supplementary Table S1.

The search results were screened for relevance to the central questions of this review, with priority given to original human studies, clinical trials, human ex vivo studies, experimental sepsis models and mechanistic studies directly informing TIM-3 biology or the interpretation of immune checkpoint signals. Non-sepsis studies were included selectively when they provided mechanistic information that was not available from sepsis-specific studies and are identified as such in the text. Studies with limited relevance regarding cell types, immune states, ligand contexts, checkpoint function or translational immunomodulation were not prioritized. Because the evidence base spans heterogeneous clinical, ex vivo, animal and mechanistic study designs, no single formal risk-of-bias scoring instrument was applied. Instead, the evidence was appraised qualitatively according to the study design, disease relevance, cohort size and setting, functional or causal perturbation, independent replication and translational relevance. Human interventional, human observational, human ex vivo functional, experimental sepsis and non-sepsis mechanistic evidence was distinguished throughout the review. Importantly, conflicting, neutral and negative findings were retained when relevant and were considered explicitly when assessing the consistency, context dependence and limitations of the available evidence.

## 3. Immune Checkpoint Pathways in Sepsis: Therapeutic Promise and Conceptual Pitfalls

Immune checkpoints entered the sepsis field with an appealing—but still incompletely proven—logic. Patients who survive the first inflammatory insult may later show lymphocyte loss, impaired antigen presentation, weak cytokine responses and poor pathogen clearance. Autopsy and peripheral blood studies gave early support to this idea, particularly for the PD-1/PD-L1 axis [16,17,18,19]. However, the clinical evidence is often not as straightforward as the therapeutic picture suggests. In a systematic review of 36 clinical studies, septic patients more often showed increased CD4+ T-cell PD-1 and monocyte PD-L1 compared with healthy controls, but the signal became less consistent when septic patients were compared with critically ill non-septic controls or when non-survivors were compared with survivors [13]. The severity of illness, comorbidities and control selection were frequent confounders. This provides an important warning. Checkpoint expression may mark immune dysfunction, but it does not by itself prove that blockade is needed.

PD-1/PD-L1 remains the most clinically developed pathway in sepsis. The strongest human interventional evidence so far is early-phase rather than efficacy-based. In a randomized, double-blind phase 1b study, 31 adults with sepsis from 10 US ICUs were enrolled at least 24 h after sepsis diagnosis, had at least one form of organ dysfunction and had lymphopenia, defined as an absolute lymphocyte count ≤ 1.1 × 10^3^ cells/μL [20]. A single dose of nivolumab, 480 mg or 960 mg, produced sustained target engagement, with >90% PD-1 receptor occupancy maintained through day 28. The median monocyte HLA-DR increased by day 14 in both dose groups, and the measured pro-inflammatory cytokines did not show an overall increase. Nevertheless, this was not a placebo-controlled efficacy trial. Twelve patients died during follow-up, serious adverse events occurred in eight patients, and immune-mediated events that were considered possibly drug-related occurred in five patients [20]. The study supports feasibility and target engagement. It does not yet indicate which septic patient should receive PD-1 blockade or when.

Other checkpoints are less mature in clinical development but biologically relevant. CTLA-4 is central to T-cell priming and regulatory T-cell biology, although the broad manipulation of this pathway in acute infection raises obvious safety concerns [21,22]. BTLA, LAG-3, TIGIT, VISTA and TIM-3 have also been reported in sepsis-related immune dysfunction, but the evidence varies by molecule, cell type and model [23,24]. TIM-3 already has direct sepsis-relevant evidence in NKT cells, where increased TIM-3 expression has been associated with apoptosis and adverse outcomes, but this signal is better viewed as a cell-specific circuit than as a general indication for blockade [15].

A second conceptual problem is the cellular source. Single-cell studies make this difficult to ignore. Reyes and colleagues profiled 106,545 PBMCs and 19,806 dendritic cells from 29 patients with sepsis and 36 controls, defining 16 immune cell states [9]. A CD14+ monocyte state, MS1, was significantly enriched in sepsis compared with controls, but it was only slightly higher when septic patients were compared with critically ill patients without infection. This is significant for checkpoint biology. A whole-blood increase in PD-L1, TIM-3 or another inhibitory molecule may reflect a shift in cell state composition, a true increase within one cell type or both. These are not equivalent therapeutic situations.

Thus, the main pitfall is not enthusiasm for checkpoint modulation. The pitfall is treating all checkpoint signals as if they carry the same meaning. In an exhausted CD8+ T cell, an inhibitory receptor may mark a reversible functional brake. In a monocyte or dendritic cell, the same pathway may reflect antigen presentation failure, damage sensing or an attempt to restrain inflammatory injury [25]. In an environment enriched in myeloid-derived suppressor cells (MDSCs), the release of T cells from PD-1 or TIM-3 may be too weak unless the suppressive myeloid network is also addressed [18,19,24]. Sepsis does not require stronger immunity in general. It requires the appropriate immune function in the appropriate compartment and at the appropriate time.

This is why checkpoint targeting in sepsis should be framed less as blanket blockade and more as immune rebalancing. PD-1/PD-L1 blockade has entered human testing and appears feasible in selected lymphopenic patients, but its efficacy remains unproven. TIM-3 provides a more complex model, with direct sepsis data in NKT cells and more indirect evidence in myeloid biology. The next question is therefore not whether a checkpoint is upregulated. It is whether the dominant checkpoint-expressing circuit in a patient is preventing immune recovery, limiting tissue damage or merely indicating that the immune system has already changed.

## 4. Phase-Dependent Effects of Immune Checkpoint Targeting in Sepsis

Timing is one of the most important variables in sepsis immunotherapy. The same intervention may be useful, irrelevant or harmful depending on the immune state in which it is given. Sepsis is diagnosed at a clinical time point, but the host response is not static. In this review, we use “immune phase” to denote a dynamic, clinically actionable host response state defined by the longitudinal balance among the inflammatory burden, innate immune competence, lymphoid effector function and suppressive immune network activity, rather than by a fixed time after diagnosis or ICU admission [2,18]. Accordingly, the terms “early hyperinflammation”, “transition”, “immunoparalysis” and “persistent remodeling” are used here as conceptual descriptions of dominant host response patterns rather than as mutually exclusive chronological stages. Individual patients may display features of more than one state simultaneously and may move between states in either direction over time.

Recent endotyping data make this definition less abstract. In the SUBSPACE/Hi-DEF analysis, which integrated transcriptomic data from more than 7000 samples across 37 cohorts, myeloid and lymphoid dysregulation scores were strongly associated with disease severity and mortality, and dysregulation on either axis identified patients with severe infection more successfully than a syndromic label alone [26]. A complementary consensus blood transcriptomics framework also supports the idea that sepsis immune states can be classified reproducibly across cohorts and may inform future precision medicine trial design [27]. Accordingly, the elapsed time from diagnosis alone should not be used to assign the immune phase.

During an inflammation-dominant state, inhibitory checkpoint signaling could represent a compensatory restraint on collateral inflammation. Innate immune activation, complement and coagulation cascades, endothelial injury and cytokine release can rise rapidly after infection and tissue damage [5,6,18]. This provides biological rationale for caution when considering checkpoint inhibition during uncontrolled inflammation. However, direct sepsis-specific evidence indicating that early TIM-3 inhibition removes a protective checkpoint brake is currently limited. Early checkpoint blockade should therefore not be assumed to be either beneficial or harmful solely on the basis of chronological timing; its effects require evaluation in relation to the contemporaneous immune state and the specific checkpoint-expressing cellular circuit.

The transition phase is likely the most actionable window, but it is also the easiest to misclassify. At this point, inflammation may still be visible, while monocyte HLA-DR falls, LPS-induced cytokine responses weaken, lymphocytes are lost or exhausted and suppressive myeloid programs begin to expand [6,28]. Protein trajectory data make this problem concrete: in a recent ICU cohort analysis summarized by Giamarellos-Bourboulis and colleagues, 345 patients were profiled with 30 immune biomarkers every 8 h for up to 7 days, and almost 65% moved between immune profiles over time [29]. A single baseline label may therefore age quickly. For checkpoint targeting, this is significant. A PD-1- or TIM-3-positive lymphocyte during immune collapse does not provide the same therapeutic signal as the equivalent marker during uncontrolled early inflammation. The TIM-3 data discussed below fit this logic: CD4 T-cell-specific TIM-3 deletion appears protective in a second-hit immunosuppressive model but not in a first-hit inflammatory model, whereas NKT and hepatic CD8+ T-cell studies suggest different kinetics across early cecal ligation and puncture (CLP).

The strongest recent clinical argument for phase-gated immunotherapy comes from ImmunoSep, although this was not a checkpoint trial. In this international, double-blind phase 2b trial, patients were treated within 72 h of sepsis onset only if they met biomarker-defined criteria for macrophage activation-like syndrome or sepsis-induced immunoparalysis. Macrophage activation-like syndrome (MALS) was defined by ferritin > 4420 ng/mL, whereas immunoparalysis required ferritin ≤ 4420 ng/mL and fewer than 5000 HLA-DR receptors on CD45/CD14 monocytes [30]. Patients with MALS received anakinra; those with immunoparalysis received recombinant interferon-γ. Precision immunotherapy increased the proportion of patients achieving the prespecified SOFA-based endpoint by day 9 and more frequently reversed immune dysfunction, but it did not significantly reduce 28-day mortality [30]. The trial therefore supports immune state-guided treatment, while also showing that immune stratification is necessary but not sufficient.

Once immunoparalysis is established, checkpoint signaling may be only one part of a wider suppressive network. Lymphocytes may already be depleted rather than simply inhibited; antigen-presenting cells may have lost HLA-DR and co-stimulatory capacity; MDSCs and regulatory cytokines may be self-sustaining [17,19,28]. In this setting, a checkpoint antibody alone may be insufficient. Older immune-restorative trials already point in this direction: granulocyte–macrophage colony-stimulating factor (GM-CSF) was tested to restore monocyte immunocompetence, and IL-7 increased lymphocyte recovery in septic shock patients with lymphopenia [31,32,33]. Earlier IFN-γ work also showed that sepsis-associated monocyte deactivation can be partially reversed, supporting the broader concept that immune paralysis is not merely descriptive but potentially targetable [34]. Late checkpoint modulation, if used at all, may need to be combined with lymphocyte support, myeloid restoration, source control and antimicrobial optimization.

Some survivors then enter persistent inflammation, immunosuppression and catabolism, with recurrent infection and long-term organ dysfunction [35]. In these patients, checkpoint expression may reflect chronic immune remodeling rather than an acutely reversible brake. The practical message is simple but difficult to execute: checkpoint targeting in sepsis should be phase-gated, not diagnosis-gated. Timing is not a technical detail. It is part of the mechanism.

Pathogen, infection-source and host modifiers of checkpoint biology should be considered. Immune state classification should also be interpreted within the broader context of the infecting organism, anatomical source and pre-existing host immune architecture. In critically ill patients with bloodstream infections, the causative pathogen accounted for a substantial proportion of blood-transcriptomic variation, with distinct host response patterns observed across organisms including Escherichia coli, Staphylococcus aureus and Streptococcus species [36]. The infection source also modifies the host response: in a prospective sepsis cohort, abdominal, respiratory, urinary, cardiovascular, central nervous system and skin infections differed in inflammatory, endothelial, coagulation and leukocyte transcriptional responses, and the infection source remained associated with the outcome after adjustment for major confounders [37]. These observations argue against assuming that an identical checkpoint phenotype has the same biological meaning across Gram-negative, Gram-positive, fungal and viral sepsis. Viral infection may also provide a distinct context; for example, altered TIM-3 expression and NKT cell depletion have been reported in SARS-CoV-2 infection [38], although direct comparative TIM-3 data across pathogen classes in sepsis remain limited.

Host factors provide a second layer of heterogeneity. Age, sex, comorbidities, prior microbial exposure and medications can modify the baseline and sepsis-induced immune response [39]. Pre-existing immunocompromise is also not immunologically uniform: in two sepsis cohorts, hematologic malignancy was strongly associated with a hyperinflammatory subphenotype, whereas the associations differed for other forms of immunocompromise [11]. Chronic liver disease provides another relevant example because cirrhosis-associated immune dysfunction is accompanied by altered lymphocyte phenotypes; peripheral CD8+ T cells from patients with cirrhosis showed increased TIM-3 expression associated with disease severity [40]. These factors should therefore be recorded as prespecified covariates or potential effect modifiers in future checkpoint studies, rather than being treated as background demographic variables. However, the current evidence is insufficient to define TIM-3-specific treatment rules according to the pathogen, infection source, age, sex or comorbidity. A comprehensive analysis of all critical illness comparators, trauma-associated immune dysfunction, immunostimulatory strategies and negative checkpoint studies is beyond the focused scope of this review; this should be recognized as a limitation when generalizing the proposed framework.

## 5. Cell Type-Dependent Effects of Immune Checkpoint Targeting in Sepsis

The effect of immune checkpoint targeting in sepsis depends not only on when the intervention is given but also on which immune cell is being influenced. While this appears overt, it is often neglected when checkpoint molecules are discussed as if they have one dominant function. A receptor that marks exhaustion in a CD8+ T cell may indicate suppressive fitness in a Treg cell, impaired phagocytosis in a macrophage, antigen-presentation failure in a monocyte or regulatory programming in a dendritic cell. A blood sample may show that PD-1, PD-L1, TIM-3 or another checkpoint is “upregulated”. This is only one aspect—the cellular source is also significant [17,18,19,41].

Effector lymphocytes are still the most intuitive targets for checkpoint-based immune restoration. Septic patients often develop lymphopenia, increased lymphocyte apoptosis, reduced cytokine production and features of T-cell exhaustion [16]. Recent reviews further emphasize that lymphopenia is not only a prognostic marker but also a central feature of sustained sepsis-associated immunosuppression and a potential enrichment criterion for immune-restorative strategies [42]. PD-1/PD-L1 signaling is the best-studied pathway in this setting; ex vivo PD-1 or PD-L1 blockade can partially restore T-cell function in selected septic patient samples [43]. However, recent human data complicate this picture. In a prospective study of 156 ICU patients and 24 healthy controls, CD4+ T-cell remodeling differed sharply by subset: in septic critical illness, conventional CD4+ T-cell subsets were reduced, whereas the Treg frequency was relatively preserved. After stimulation, CD4+ Tconv cells showed increased CD25, PD-1 and LAG-3—a mixed phenotype of activation and impairment. Tregs showed higher PD-1 and LAG-3 and sustained CD39 and TIGIT expression, consistent with a more suppressive state [44]. A checkpoint-positive T cell in sepsis may therefore be exhausted and worth rescuing, but it may also be a suppressive Treg that the host has kept alive.

NK and NKT cells add another layer because they lie near the boundary between innate and adaptive immunity. This is particularly relevant for unconventional T-cell populations, whose roles in sepsis are increasingly recognized but remain difficult to translate into uniform therapeutic rules [45]. Experimental CLP studies provide preclinical functional evidence that the perturbation of the TIM-3/Galectin-9-associated axis alters NKT cell apoptosis and survival [46], whereas the relevant human data are observational, showing associations between TIM-3+ NKT cells, apoptosis, disease severity and 28-day mortality [15]. Taken together, these findings support a sepsis-relevant TIM-3/NKT cell axis but do not establish the therapeutic efficacy of TIM-3-directed intervention in patients.

A sepsis study examining the CD8+ T-cell compartment added a tissue-specific dimension: septic patients showed higher TIM-3 on peripheral CD8+ T cells, whereas CLP mice showed dynamic hepatic CD8+ T-cell TIM-3 expression and reduced liver T-cell apoptosis after TIM-3 mutation or α-lactose treatment [47]. This is an important nuance. TIM-3-directed intervention may rescue lymphoid cells in one tissue compartment, while its systemic effect will still depend on the action of TIM-3 in other cells at the same time.

Myeloid cells require a different reading. Monocytes and macrophages in sepsis can lose HLA-DR, reduce inducible cytokine production and fail to clear pathogens, while still contributing to organ inflammation [19,41]. Checkpoint molecules in these cells therefore may not indicate “exhaustion” in the T-cell sense. A recent CLP exosome study illustrates this point. Exosomes isolated from septic mice inhibited macrophage proliferation by about 40%, increased apoptosis 1.67-fold, suppressed M2 polarization, upregulated macrophage PD-1 and impaired phagocytosis; co-culture experiments then linked exosome-conditioned macrophages to Th17-skewed T-cell responses [48]. This is mouse-model evidence, not a clinical checkpoint trial. Nonetheless, it shows why checkpoint biology in sepsis must include macrophage dysfunction and altered T-cell instruction, not only lymphocyte exhaustion.

Dendritic cells are equally difficult to classify. In a multi-compartment single-cell study of murine polymicrobial sepsis, investigators identified a sepsis-induced mature regulatory DC population with high PD-L1 and CD80 expression. These mregDCs increased after CLP, reached about 15% of splenic DCs at 24 h and expressed maturation and migration markers [49]. Functionally, sorted mregDCs induced stronger naïve CD4+ T-cell activation and proliferation than non-mregDCs, but they also promoted IL-10 production and Treg differentiation [49]. Therefore, a PD-L1-high DC is not automatically negative. It may be attempting to activate, restrain and redirect T-cell responses at the same time. Although these examples focus mainly on PD-1/PD-L1 biology, they illustrate the same point that applies to TIM-3: receptor expression cannot be interpreted without knowing the cell state that carries it.

The late suppressive myeloid compartment is another reason that single-cell-source thinking is important. In a retrospective cohort of 301 septic shock patients, CD14+HLA-DR-low monocytic MDSC-like cells were markedly increased compared with healthy controls. Early values were not strongly informative, but persistence at days 6–8 was associated with 28-day mortality and ICU-acquired infection; the adjusted odds ratios were 4.4 for mortality and 2.4 for nosocomial infection [50]. If this network dominates, the release of T cells from PD-1 or TIM-3 inhibition may be too weak unless suppressive myeloid activity is also addressed [51,52].

The practical conclusion poses a challenge but is necessary: in sepsis, checkpoint targeting cannot be interpreted at the level of the molecule alone. The relevant unit is the checkpoint-expressing cell circuit. It is possible to block the correct molecule in the wrong cell.

## 6. TIM-3 as a Model of Cell Type- and Phase-Dependent Checkpoint Biology

TIM-3 is a useful checkpoint to consider precisely because it refuses to behave as a single-purpose inhibitory receptor. It was originally described on Th1 cells as a regulator of inflammatory T-cell responses [53]. Later work placed it among co-inhibitory receptors such as PD-1, LAG-3 and TIGIT, although recent checkpoint biology emphasizes that these receptors have distinct and context-specific functions rather than interchangeable inhibitory roles [54,55]. This classification is convenient, but, in sepsis, it is too narrow. TIM-3 is expressed on CD4+ and CD8+ T cells, NKT cells, NK cells, monocytes, macrophages, dendritic cells and some tissue-resident myeloid cells [38,56,57]. It also binds several ligands, including Galectin-9, carcinoembryonic antigen-related cell adhesion molecule 1 (CEACAM1), high-mobility group box 1 (HMGB1) and phosphatidylserine [58,59,60,61]. A receptor with this type of cellular and ligand distribution is unlikely to have one stable meaning during a disease that is as dynamic as sepsis. Its ligand network is one reason for this instability, and this is discussed in the next section.

The evidence base is uneven, and this should be noted clearly. The most direct sepsis-specific data involve lymphoid cells, especially NKT cells and CD8+ T cells. Evidence for myeloid TIM-3 in sepsis is more fragmentary and often inferred from studies of dendritic cells, macrophages, microglia, transplantation or tumor immunity. This unevenness positions TIM-3 as a more faithful model of checkpoint complexity. TIM-3 is not useful here because everything is already settled; it is useful because it exposes where the cell type, disease phase, ligand and tissue compartment change the interpretation of a checkpoint signal [62].

One series of experimental and clinical studies provides sepsis-relevant evidence for TIM-3 cell specificity. In a CLP model, TIM-3 expression on NKT cells increased rapidly after sepsis and peaked at approximately 24 h; dendritic cell-derived IL-12 promoted this increase, whereas IL-12 deficiency reduced TIM-3 expression and NKT cell apoptosis [46]. α-Lactose, used to interfere with the TIM-3/Galectin-9-related axis, reduced NKT cell apoptosis, dampened inflammatory cytokine responses and improved survival in septic mice [46]. A subsequent single-center clinical and experimental study found increased TIM-3 expression on NKT cells in patients with sepsis, with associations between TIM-3+ NKT cells, NKT cell apoptosis, disease severity and 28-day mortality [15]. In parallel, Tim-3-mutant mice showed improved survival after CLP, and α-lactose reduced mortality in wild-type septic mice [15]. Together, these observations nominate TIM-3+ NKT cells as a candidate sepsis-relevant circuit, but they do not establish the clinical efficacy of TIM-3 blockade or demonstrate that this phenotype is conserved across independent patient cohorts.

A related study suggested that TIM-3 may operate differently within the CD8+ T-cell compartment. In 35 patients with sepsis and 20 healthy controls, peripheral CD8+ T cells from patients with sepsis showed higher TIM-3 expression and increased Annexin V staining [47]. The stronger mechanistic evidence comes from experimental sepsis: in CLP mice, hepatic CD8+ T-cell TIM-3 expression displayed a biphasic pattern, hepatic CD8+ T-cell apoptosis peaked at approximately 36 h, and Tim-3-mutant mice showed reduced liver T-cell apoptosis. α-Lactose also reduced TIM-3 expression at selected time points and attenuated liver inflammation and injury [47]. Independent experimental work has provided partial support for the broader CD8+ T-cell phenotype. Liu et al. reported increased TIM-3 expression on splenic CD8+ T cells after CLP and found that anti-TIM-3 antibody treatment reduced lymphocyte apoptosis and attenuated sepsis severity in mice [63]. However, this study did not reproduce the liver-specific biphasic TIM-3 kinetics or the hepatic CD8+ T-cell phenotype described above. Thus, independent evidence supports a broader relationship between TIM-3 and CD8+ T-cell dysfunction in experimental sepsis, whereas the liver-specific phenotype remains to be independently validated.

These findings should nevertheless be interpreted in light of several important limitations. The human NKT cell study included 55 patients with sepsis or septic shock and 26 healthy controls, whereas the CD8+ T-cell study included 35 patients with sepsis and 20 healthy controls [15,47]. Both clinical components were derived from single-center cohorts and were primarily observational, which limits generalizability and precludes firm causal inference in human sepsis. In addition, the NKT cell phenotype has not, to our knowledge, been independently replicated in an external clinical cohort. For CD8+ T cells, independent experimental work supports a broader association between TIM-3 signaling, lymphocyte apoptosis and sepsis severity [63], but the liver-specific biphasic TIM-3 expression pattern and hepatic CD8+ T-cell apoptosis described in the original study have not been independently reproduced. The NKT and hepatic CD8+ T-cell findings should therefore be regarded as mechanistically informative and hypothesis-generating rather than as established, independently validated features of human sepsis.

CD4+ T-cell data illustrate the phase issue more clearly. Ex vivo TIM-3 and PD-1 blockade increased cytokine secretion from lymphocytes and monocytes isolated from patients with sepsis, demonstrating short-term functional reversibility in vitro, rather than in vivo therapeutic efficacy [64]. In a separate study, increased TIM-3 expression on CD4+ T cells was observed in patients with sepsis-associated immunosuppression, whereas cell-specific causal evidence came from mice: CD4+ T-cell Tim-3 deletion improved survival in a CLP second-hit model but not in first-hit CLP [65]. The same experimental work linked HMGB1-TIM-3 signaling to reduced NF-κB/TNF-α activation [65]. These model-dependent effects support an immune state-specific interpretation of TIM-3 function.

Experimental studies have also reported divergent effects of TIM-3-associated interventions. Galectin-9 administration prolonged survival in CLP mice; decreased TNF-α, IL-6, IL-10 and HMGB1; and increased IL-15 and IL-17, while expanding TIM-3-expressing NKT cells and PDCA-1+CD11c+ macrophage-like cells [66]. This does not simply contradict the α-lactose data. Recombinant Galectin-9 administration, Galectin–glycan interference, receptor mutation and antibody blockade do not perturb the same biology. A similar caution emerges from non-sepsis transplantation and infection models, where TIM-3 can support tissue restraint or host defense rather than simply suppressing immunity [67,68,69,70].

**Figure 2:**
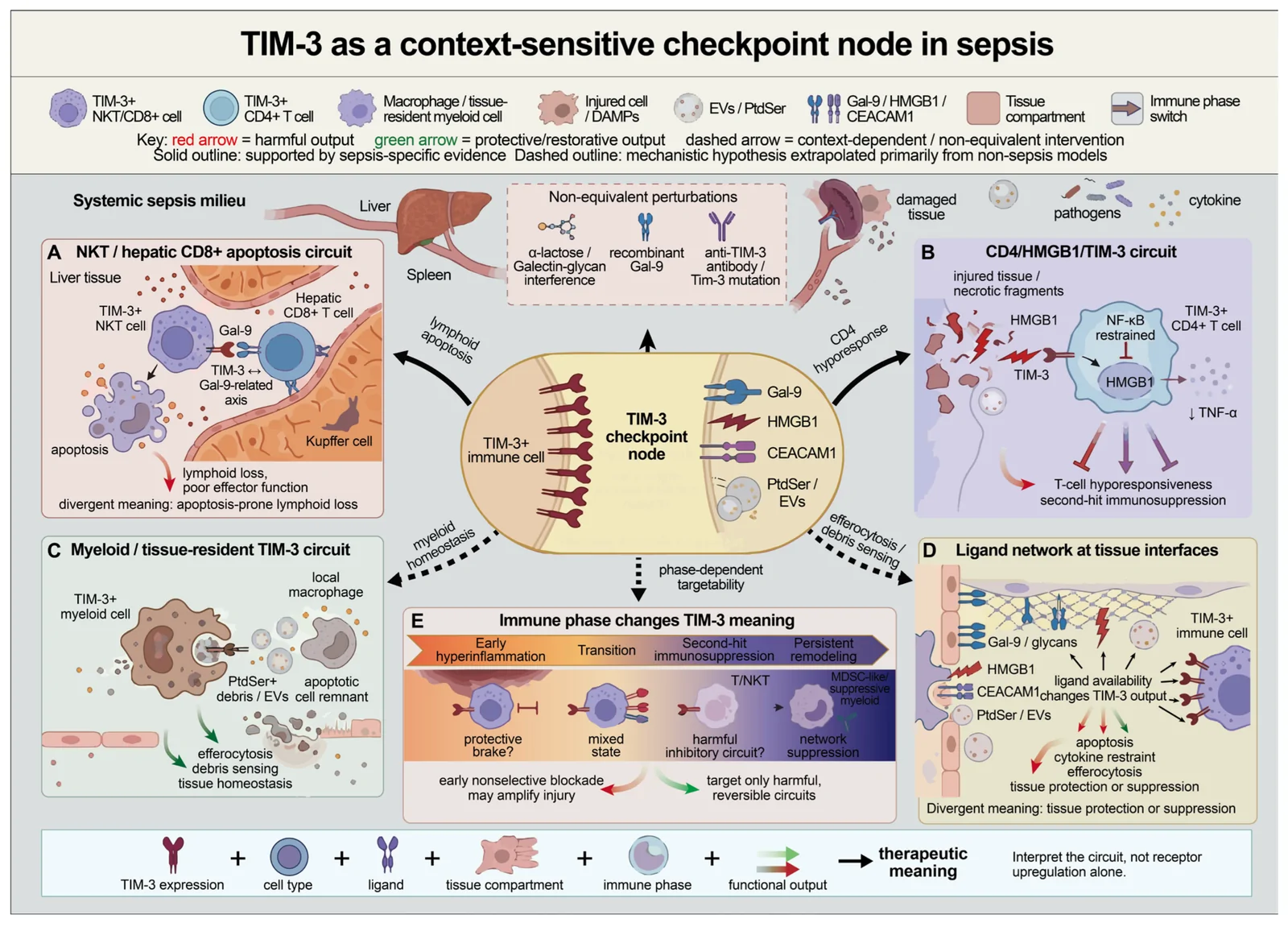
TIM-3 as a context-sensitive checkpoint node in sepsis. TIM-3 should be interpreted as a context-sensitive checkpoint node rather than as a single-purpose inhibitory receptor in sepsis. Its biological meaning depends on the checkpoint-expressing cell type, ligand availability, tissue compartment and immune phase. In sepsis-relevant settings, TIM-3-associated circuits may involve NKT cell or hepatic CD8+ T-cell apoptosis through Galectin-9-related interactions; CD4/HMGB1/TIM-3-associated hyporesponsiveness with restrained NF-κB and TNF-α signaling; myeloid or tissue-resident interactions with PtdSer-positive debris and extracellular vesicles; and ligand-rich tissue interfaces involving Galectin-9, HMGB1, CEACAM1 and phosphatidylserine. These circuits may generate divergent functional outputs, including apoptosis, cytokine restraint, efferocytosis, tissue protection or immunosuppression. Therefore, TIM-3 targetability should be inferred from a defined immune circuit rather than receptor upregulation alone.

TIM-3 therefore should not be presented simply as another inhibitory receptor waiting to be blocked. A better description is that TIM-3 is a context-sensitive checkpoint node (Figure 2). In NKT cells and hepatic CD8+ T cells during experimental and clinical sepsis, the available studies support an association between TIM-3, apoptosis and immune injury, although the extent of independent replication differs between these phenotypes. In CD4+ T cells during immunosuppression, HMGB1-TIM-3 signaling may restrain NF-κB/TNF-α responses. In myeloid and tissue-resident compartments, TIM-3 may participate in danger sensing, efferocytosis, inflammasome regulation or tissue homeostasis, as suggested by non-sepsis studies linking TIM-3 to inflammasome control and tissue immune restraint [71]. This is why TIM-3 is a useful model for precision immunomodulation in sepsis: it forces the therapeutic question to become more specific—which TIM-3-dependent circuit, in which cell, at which phase and under which ligand or tissue context?

## 7. TIM-3 Ligand and Microenvironmental Context: Why the Same Checkpoint Signal Changes Meaning

TIM-3 biology becomes difficult to interpret once its ligands are considered. It is tempting to draw a simple receptor–ligand diagram: TIM-3 on the immune cell and Galectin-9 or another ligand on the other side, followed by inhibition. However, such a simplified receptor–ligand model may be misleading. TIM-3 can interact with Galectin-9, CEACAM1, HMGB1 and phosphatidylserine, and these ligands do not appear in the same place, at the same time or on the same molecular platform [58,59,60,61]. In sepsis, cell death, DAMP release, microbial products, endothelial injury, extracellular vesicles and tissue hypoxia all change the ligand field around TIM-3. A TIM-3+ NKT cell exposed to a Galectin-9-rich inflammatory milieu is not equivalent to a TIM-3+ CD4 T cell encountering HMGB1 during immunoparalysis or to a TIM-3+ macrophage handling apoptotic cell debris in an injured liver. Accordingly, identical TIM-3 expression does not imply equivalent biological output across these contexts.

Galectin-9 is the best-known example, and it is also the most difficult to interpret. In the canonical T-cell literature, Galectin-9–TIM-3 engagement can induce the apoptosis of TIM-3+ Th1 cells and restrain inflammatory T-cell responses [58]. In sepsis models, however, Galectin-9-related interventions have not pointed in one direction. One CLP study showed that α-lactose, used to interfere with the TIM-3/Galectin-9-related axis, reduced NKT cell apoptosis and improved survival in CLP mice [46]. However, Kadowaki and colleagues reported that recombinant Galectin-9 administration also prolonged survival in septic mice; reduced plasma TNF-α, IL-6, IL-10 and HMGB1; and expanded TIM-3-expressing NKT cells together with PDCA-1+CD11c+ macrophage-like cells [66]. This apparent paradox should not be interpreted as evidence that the opposite manipulation of a single linear TIM-3 pathway is equally beneficial. Rather, recombinant ligand administration, Galectin–glycan interference, receptor mutation and antibody blockade perturb partially overlapping but biologically distinct signaling networks. α-Lactose, in particular, is not a TIM-3-specific drug. It can affect broader Galectin–glycan interactions. The ligand context is therefore not a technical footnote; it is part of the mechanism.

One testable explanation for these apparently convergent survival effects is that α-lactose and recombinant Galectin-9 do not represent opposite perturbations of the same linear TIM-3 pathway. α-Lactose broadly interferes with Galectin–glycan interactions rather than selectively antagonizing TIM-3, whereas exogenous Galectin-9 can engage TIM-3 as well as other glycoprotein receptors, with the dominant functional output varying by cell type, ligand concentration and immune state [46,47,72]. Thus, Galectin-9 administration during an inflammatory phase could reduce injurious inflammation through regulatory lymphoid or myeloid effects, whereas α-lactose could simultaneously be beneficial in a different cellular circuit by limiting Galectin-associated pro-apoptotic signaling in NKT or CD8+ T cells. These possibilities remain hypotheses rather than established explanations. They could be distinguished experimentally by head-to-head dose- and timing-matched comparisons of recombinant Galectin-9, α-lactose and a selective anti-TIM-3 antibody within the same sepsis model; by repeating these interventions in lineage-specific Havcr2/Tim-3 conditional models; and by Galectin-9 neutralization and add-back experiments in wild-type and TIM-3-deficient backgrounds. Parallel measurement of lymphocyte apoptosis, myeloid function, cytokine responses, bacterial clearance, organ injury and survival would determine whether the interventions converge on a common TIM-3-dependent mechanism or achieve similar outcomes through distinct cellular pathways.

Recent ligand comparison work in NK cells further illustrates the receptor-specific complexity. In a 2025 study using human NK cells and head and neck squamous cell carcinoma models, Wang and colleagues compared four putative TIM-3 ligands and found that Galectin-9 most consistently suppressed NK cell cytotoxicity and proliferation [72]. The underlying receptor dependence differed by functional endpoint: the suppression of cytotoxicity was TIM-3-dependent, whereas the inhibition of proliferation also involved CD44, and the available TIM-3 antagonists did not fully reverse the Galectin-9-mediated effects [72]. Because this study was performed outside sepsis, these findings should not be taken as evidence that the same receptor redundancy occurs in septic NK or NKT cells. Rather, they provide mechanistic rationale for testing whether Galectin-9 signals through TIM-3-independent pathways in sepsis. Whether such redundancy limits the functional effects of TIM-3 blockade in septic NK or NKT cells remains unknown.

HMGB1 adds another layer because it is both a TIM-3 ligand and a DAMP released during tissue injury, necrosis and inflammatory stress. In dendritic cells, TIM-3 can bind HMGB1 and prevent HMGB1-mediated nucleic acid sensing, thereby dampening innate immune activation [60]. In sepsis-induced immunosuppression, HMGB1 may also operate in T cells. In a study of CD4 T cells during septic immunosuppression, Galectin-9 did not behave as the functional TIM-3 ligand. Instead, HMGB1 bound TIM-3 with relatively strong affinity, colocalized with TIM-3 in LPS-treated TIM-3+ CD4 T cells and was linked to the inhibition of NF-κB/TNF-α signaling [65]. The same study showed that TIM-3 deletion in CD4+ T cells was protective in a CLP second-hit immunosuppressive model but not in the first-hit inflammatory model [65]. Ligand identity, therefore, cannot be separated from the disease phase.

CEACAM1 brings the tissue interface into the discussion. In T cells, CEACAM1 can cooperate with TIM-3 to regulate tolerance and exhaustion [59]. A translational liver transplantation study further showed that CD4+ T-cell CEACAM1 promoted TIM-3 signaling and, through interaction with hepatic CEACAM1, suppressed NF-κB activation in Kupffer cells [67]. This was not a sepsis experiment, but it is mechanistically useful. It shows that TIM-3 signaling can be an intercellular conversation at a tissue boundary, not simply an inhibitory mark on an isolated leukocyte.

Phosphatidylserine (PtdSer) is also relevant to the septic environment. Apoptotic cells, platelet-derived vesicles, endothelial microparticles and injured tissue debris can all expose phosphatidylserine. TIM-family receptors use phosphatidylserine recognition to participate in apoptotic cell clearance and immune regulation [61]. A photopharmacology study using a photoswitchable phosphatidylserine ligand showed that controlled PtdSer-TIM-3 engagement could suppress NK cell activation and reduce liver inflammation in an acute hepatitis model [73]. The model was artificial, but it proves a useful principle: the spatial and temporal presentation of a TIM-3 ligand can determine immune output.

For sepsis studies, ligand assessment should therefore be integrated with the cell identity, tissue compartment, immune state and functional readouts. Experimental designs should determine whether candidate ligands are present in the same compartment as TIM-3-expressing cells and whether their presence is associated with specific outcomes, such as apoptosis, cytokine suppression, impaired antigen presentation, efferocytosis or tissue protection. Such paired measurements would help to distinguish ligand-dependent mechanisms from changes in receptor abundance and provide a stronger basis for functional perturbation studies.

## 8. TIM-3-Informed Precision Immunomodulation in Sepsis

Precision immunomodulation in sepsis should be framed as a translational research strategy rather than as evidence that TIM-3 is ready for clinical targeting. Sepsis-associated immune dysregulation is heterogeneous and dynamic [7,18], and the current TIM-3 evidence base spans substantially different levels of inference. Human studies are largely observational or ex vivo, whereas stronger causal evidence for specific TIM-3-associated phenotypes comes predominantly from CLP, receptor mutant or cell-specific knockout models [46,47,65]. The immediate objective should therefore be to define reproducible TIM-3-associated circuits in patients using prespecified criteria and determine whether these circuits are causally involved and functionally reversible before TIM-3-directed intervention is advanced as a therapeutic strategy.

The broader sepsis field nevertheless provides proof of principle that immune state-guided intervention is feasible. ImmunoSep is particularly informative, although it was not a checkpoint trial. Patients were assigned to macrophage activation-like syndrome or sepsis-induced immunoparalysis using predefined biomarkers and received matched treatment with anakinra or recombinant IFN-γ. The precision immunotherapy strategy improved the prespecified SOFA-based primary endpoint but did not significantly reduce 28-day mortality [30]. Thus, ImmunoSep supports the feasibility of biomarker-guided immunomodulation while also showing that biological stratification alone does not establish efficacy for any specific immune pathway or checkpoint target.

A TIM-3-oriented translational framework could integrate four linked domains. The first is the inflammatory burden, including ferritin, IL-6, the shock context, the SOFA trajectory and organ injury. The second is innate immune competence, including monocyte HLA-DR and inducible cytokine responses, which are established measures of sepsis-associated immune dysfunction [19]. The third is the lymphoid status, including absolute lymphocyte counts, the CD4/CD8/NK/NKT cell composition, apoptosis, IFN-γ production and checkpoint co-expression. Importantly, early clinical checkpoint studies in sepsis have primarily established feasibility, safety, pharmacokinetics/pharmacodynamics or functional reversibility rather than clinical efficacy [20,64]. The fourth is the ligand context. Galectin-9, HMGB1, CEACAM1 and phosphatidylserine should be considered when experimentally feasible because they engage distinct aspects of TIM-3 biology [58,59,60,61]. At present, these measurements should be viewed as components of a research framework for circuit definition, rather than as a validated clinical selection algorithm.

These four domains should be interpreted jointly rather than as independent binary biomarkers. Prospectively, patients could be provisionally characterized according to the dominant pattern of immune dysfunction. A high inflammatory burden with relatively preserved innate and lymphoid competence would indicate an inflammation-dominant state; reduced monocyte HLA-DR and inducible cytokine responses would support an innate immunoparalytic state; prominent lymphopenia, apoptosis or impaired lymphocyte effector function would favor a lymphoid dysfunction-dominant state; and concurrent innate and lymphoid dysfunction together with persistent suppressive myeloid expansion would indicate a mixed suppressive state. These categories are intended as a conceptual stratification framework rather than mutually exclusive or clinically validated endotypes, because inflammatory and immunosuppressive programs frequently coexist in sepsis [18,26]. Initial classification should therefore remain provisional and be reassessed longitudinally as the host response trajectory evolves [26,29].

Operationally, circuit assignment should be hierarchical rather than enforcing a single category. A core panel of routinely or rapidly obtainable measures—including ferritin/IL-6, absolute lymphocyte counts, quantitative monocytic human leukocyte antigen DR (mHLA-DR) and, where feasible, inducible cytokine responses—would first define the contemporaneous host response state [19,28,30,42]. TIM-3-specific classification would then require concordance among cell-specific TIM-3 expression, a biologically plausible ligand context and a matched functional defect [58,59,60,61]. If two or more host response patterns coexist without a clear functional predominance, the patient should be classified as mixed; discordant profiles should remain indeterminate rather than being forced into a dominant circuit. For prospective validation, classification rules should be prespecified before outcome analysis and tested for inter-site reproducibility, temporal stability, concordance with functional assays and incremental value over simpler biomarkers. Repeated sampling should prioritize the core panel, whereas research-grade ligand assays may be performed in batches and should not currently delay enrollment decisions (Table 1).

**Figure 3:**
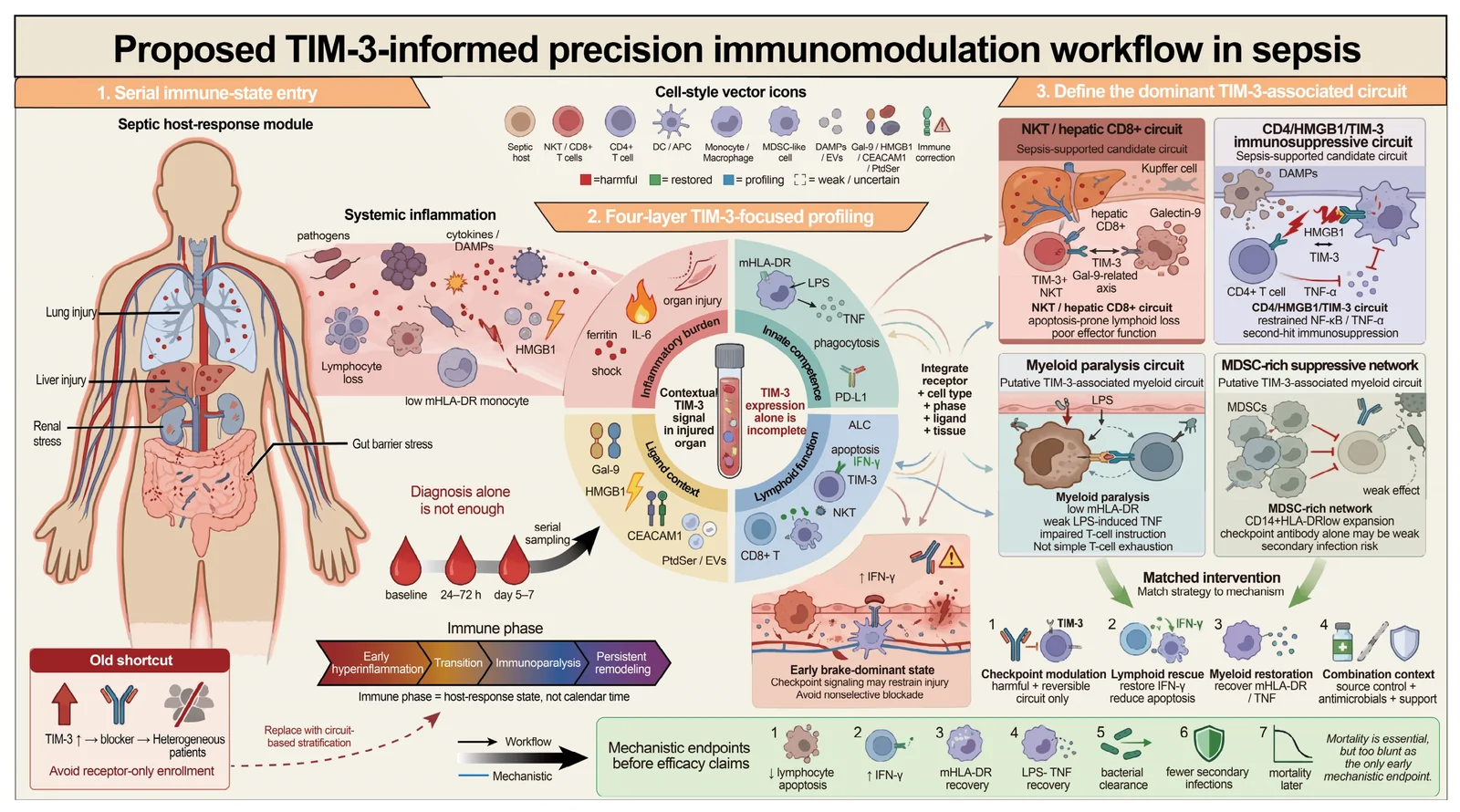
Proposed TIM-3-informed precision immunomodulation workflow in sepsis. TIM-3-informed precision immunomodulation should begin with immune circuit definition rather than receptor expression or drug selection alone. Patients with sepsis should undergo serial immune state assessment across dynamic host response phases, supported by the four-layer profiling of the inflammatory burden, innate immune competence, lymphoid function and ligand context. These data can be integrated to define dominant TIM-3-associated circuits, including NKT cell or hepatic CD8+ T-cell apoptosis, CD4/HMGB1/TIM-3-associated immunosuppression, myeloid paralysis, MDSC-rich suppression or an early hyperinflammatory brake-dominant state in which non-selective checkpoint blockade may be inappropriate. Interventions should then be matched to the dominant circuit and evaluated first by mechanistic endpoints, such as reduced lymphocyte apoptosis, restored IFN-γ production, mHLA-DR recovery, LPS-induced TNF recovery, bacterial clearance and secondary infection, before mortality is used as the principal efficacy endpoint in later trials. Solid elements indicate circuits supported directly by human or experimental sepsis studies, whereas dashed elements indicate mechanistic hypotheses extrapolated primarily from non-sepsis systems. Sampling time points are used for longitudinal reassessment and do not themselves define the immune phase; phase assignment is based on the immune profile measured at each time point.

TIM-3-specific assessment would constitute a second layer rather than the primary basis for immune state classification. Once the dominant or mixed host response state has been defined, cell-specific TIM-3 expression can be interpreted together with the ligand context—including Galectin-9, HMGB1, CEACAM1 and phosphatidylserine—and the tissue compartment and functional readouts [58,59,60,61]. This two-step approach separates global immune state classification from checkpoint-specific circuit identification and reduces the risk of assigning therapeutic meaning to TIM-3 expression in isolation. This hierarchical workflow, from serial immune-state assessment and candidate circuit assignment to matched intervention and mechanistic validation, is summarized in Figure 3.

Within such a framework, TIM-3+ NKT cell and hepatic CD8+ T-cell apoptosis can be regarded as candidate circuits supported by a combination of human observational and experimental sepsis evidence [15,46,47]. The CD4+ T-cell/HMGB1/TIM-3 pathway during sepsis-associated immunosuppression has cell-specific causal support from a second-hit mouse model, whereas the corresponding human evidence remains primarily observational [65]. By contrast, putative myeloid TIM-3 circuits are supported largely by mechanistic studies outside sepsis and require direct validation in human sepsis before therapeutic implications can be assigned. These distinctions argue against selecting patients for TIM-3-directed treatment on the basis of receptor expression alone.

Importantly, this framework should be treated as a falsifiable hypothesis rather than a self-validating classification system. Its validity would be weakened if prespecified TIM-3-associated circuits cannot be reproducibly identified across independent cohorts, if circuit assignment does not correspond to the predicted functional immune defect or if biological responses to TIM-3-directed perturbation are unrelated to the assigned circuit. The framework should also demonstrate incremental value over simpler and clinically accessible biomarkers, such as absolute lymphocyte counts and monocyte HLA-DR, which already capture important aspects of lymphoid and innate immune dysfunction in sepsis [19,42]. A circuit-based approach would be justified only if it provides reproducible information beyond these simpler measures—for example, by improving the identification of reversible immune dysfunction, risk stratification for secondary infection or the prediction of the response to a defined immunomodulatory intervention [26,28,30]. At present, the prevalence of individual TIM-3-associated circuits in unselected sepsis populations is unknown because standardized definitions and prospective multicenter measurements are lacking. Their frequency, temporal stability and association with treatment response should therefore be considered explicit validation endpoints rather than assumptions of the framework [26,29].

Accordingly, the next step should be prospective translational validation rather than an assumption of therapeutic readiness. Future prospective studies should use prespecified circuit definitions and combine serial immune state profiling with cell-specific TIM-3 measurement, ligand assessment and functional testing at predefined time points. These predefined sampling windows are intended to standardize measurement rather than to assign immune phases by clock time; phase/state assignment should be performed independently at each sampling point from the contemporaneous inflammatory, innate, lymphoid and suppressive myeloid profile. If an interventional TIM-3 study subsequently becomes justified, early endpoints should emphasize target engagement and the correction of the predefined immune defect, such as changes in lymphocyte apoptosis or inducible cytokine function, while microbiological outcomes, secondary infection, organ dysfunction and mortality remain necessary downstream clinical outcomes. On the basis of the current evidence, TIM-3 should therefore be considered a candidate for mechanistically stratified investigation in sepsis, rather than as a clinically validated therapeutic target.

## 9. Methodological Challenges and Future Directions for TIM-3-Centered Checkpoint Studies

TIM-3 expression has been documented in human sepsis samples and experimental models, but its functional meaning and causal importance remain context-dependent. The more difficult task is to determine what a TIM-3 signal means in a specific patient sample and whether it identifies a reversible, therapeutically relevant dysfunction. A higher percentage of TIM-3+ cells may reflect true receptor induction within a cell type, the expansion of a TIM-3-rich subset, the migration of TIM-3-negative cells out of the blood or the selective survival of regulatory cells. These possibilities lead to different therapeutic conclusions. An additional measurement consideration is the distinction between cell-associated and soluble TIM-3 (sTIM-3). Most sepsis studies discussed above quantify TIM-3 on defined leukocyte populations by flow cytometry, whereas circulating sTIM-3 represents an analytically distinct readout and should not be interpreted as a surrogate for cell-surface receptor abundance. In a clinical study of 100 patients classified using pre-Sepsis-3 criteria, Ren et al. measured monocyte TIM-3 by flow cytometry and plasma sTIM-3 by ELISA and observed non-parallel changes across disease severity groups. Plasma sTIM-3 was reduced in sepsis and severe sepsis but increased in septic shock associated with marked immunosuppression, and sTIM-3 levels were inversely correlated with IL-12 and TNF-α [75]. TIM-3 can also undergo proteolytic shedding involving ADAM10 and ADAM17, further separating circulating sTIM-3 from membrane receptor abundance. Endogenous sTIM-3 should additionally be distinguished from recombinant soluble TIM-3-Ig fusion proteins used experimentally to block TIM-3 signaling. Future studies should therefore report cell-associated and soluble TIM-3 as distinct variables and, where feasible, assess both longitudinally.

The first problem is immune phase annotation. “Early” and “late” sepsis are still too often defined by the clock time. This is convenient for sampling schedules, but it does not reflect immunology. Transcriptomics and protein endotyping studies show that patients can move between immune states during ICU hospitalization, and broad immune dysregulation is better captured by myeloid and lymphoid axes than by a single diagnosis [29]. TIM-3 studies should therefore report the time after sepsis onset, but they should also report immune state markers such as mHLA-DR, inducible cytokine function, lymphocyte counts, apoptosis, checkpoint co-expression and suppressive myeloid activity. A TIM-3 result without immune state annotation is difficult to interpret.

The second problem is cell definition and gating. NKT cells, NK cells, CD8+ T cells, CD4+ T cells and myeloid cells are not interchangeable TIM-3 carriers. Multi-omics analyses of neutrophil heterogeneity in sepsis further illustrate why broad lineage labels are insufficient for interpreting immune dysfunction [76]. Even within one compartment, gating choices are significant. NKT cells may be defined differently across studies; monocytic MDSC-like cells may overlap with CD14+HLA-DR-low monocytes; and dendritic cell subsets are easily distorted by tissue processing. The sepsis mregDC study illustrates a stronger design: single-cell RNA sequencing identified a regulatory DC program after CLP, but flow cytometry, tissue staining and T-cell co-culture were also used to show that PD-L1-high mregDCs could activate naïve CD4+ T cells while promoting IL-10 production and Treg differentiation [49]. TIM-3 studies should incorporate this type of discovery-plus-function logic more often.

The third problem is ligand measurement. TIM-3 should not be studied without Galectin-9, HMGB1, CEACAM1 and phosphatidylserine when feasible. Each ligand brings technical uncertainty. Galectin-9 assays may measure the total soluble protein without identifying the glycan-bound or cell-associated pool that is biologically relevant. HMGB1 can reflect necrosis, active secretion or sample handling. Phosphatidylserine may appear on apoptotic cells, platelets, endothelial vesicles or tissue debris. CEACAM1 is strongly shaped by the tissue interface. Pre-analytical handling, including the anticoagulant choice, processing delays, platelet contamination and freeze–thaw cycles, should be reported. A blood-only TIM-3 study may therefore miss the actual interaction site.

The fourth problem is intervention model equivalence. An anti-TIM-3 antibody, recombinant Galectin-9, α-lactose, ligand neutralization, global Tim-3 knockout and cell-specific TIM-3 deletion do not test the same biology. The α-lactose studies are informative because they identify Galectin-related TIM-3-associated circuits in NKT and CD8+ T cells, but α-lactose is not a selective TIM-3 blocker [46,47]. Conversely, recombinant Galectin-9 cannot be assumed to represent selective TIM-3 agonism because Galectin-9 can engage additional receptors and produce function-specific effects that are not fully reversed by TIM-3 antagonism [66,72]. Cell-specific Tim-3 deletion provides a different level of evidence, closer to causality for a defined cell compartment and disease state [65], whereas global knockout may introduce developmental or compensatory effects. Antibody blockade introduces additional pharmacological variables, including epitope, Fc function, dose, receptor occupancy, ligand competition and the duration of target engagement. Resolving apparently discordant results will therefore require head-to-head experiments that control the intervention timing and dose, incorporate cell-specific conditional models and ligand neutralization/add-back and measure both target engagement and cell-specific functional outcomes. These distinctions should be reported explicitly. Although an anti-TIM-3 antibody has shown a proof-of-concept benefit in a murine CLP study [63], to our knowledge, no TIM-3-selective agent has yet been validated in sepsis within a translationally rigorous development framework incorporating dose-ranging, PK/PD or receptor occupancy assessment, clinically relevant delayed therapeutic administration and prospective immune state selection.

The fifth problem is functional validation beyond omics. Single-cell RNA sequencing can identify TIM-3-expressing states, spatial profiling can determine whether TIM-3+ cells are located near candidate ligand-producing cells, and ligand–receptor inference can generate mechanistic hypotheses. Spatial and multi-omics studies outside sepsis further illustrate how cell state and tissue context information can nominate TIM-3 as a candidate pathway [77], but such associations do not establish causal function in sepsis. Spatial or single-cell associations should therefore be regarded as hypothesis-generating until the inferred circuit is confirmed by functional perturbation. Ex vivo receptor blockade, ligand add-back, conditional knockout, adoptive transfer experiments, bacterial clearance assays, antigen presentation assays and cytokine restimulation studies remain important approaches for establishing whether an inferred TIM-3 circuit is functionally relevant.

There are also translational pharmacology, safety and regulatory considerations. Several TIM-3-directed monoclonal antibodies have already entered clinical development in oncology, providing useful information about human pharmacology but not direct evidence for their use in sepsis. Representative agents include sabatolimab (MBG453), LY3321367, INCAGN02390 and S095018/Sym023 [70,78,79,80]. In early-phase cancer studies, the maximum tolerated doses were generally not reached, and commonly reported treatment-related events included fatigue, pruritus or rash and infusion-related reactions [78,79,80]. LY3321367 also demonstrated dose-dependent exposure and pharmacodynamic target engagement, whereas INCAGN02390 was associated with treatment-related adverse events in 30% of patients, grade ≥ 3 treatment-related events in 8% and investigator- or sponsor-defined immune-related events in approximately 10% [79,80]. Combination treatment with S095018/Sym023 and anti-PD-1 therapy was also generally tolerable, although immune-mediated toxicities including colitis and cytopenia occurred, and attribution to TIM-3 blockade alone is not possible in a combination regimen [70].

These oncology safety data cannot be directly extrapolated to sepsis. Patients with sepsis have active infections, rapidly changing immune states and frequent multiorgan dysfunction, and the removal of a protective checkpoint signal at the wrong phase could theoretically amplify inflammation or immune-mediated tissue injury. Infusion reactions, autoimmune or other immune-related toxicities and excessive inflammatory activation would therefore require prospective monitoring; importantly, cytokine amplification should be regarded as a potential sepsis-specific concern rather than an established class toxicity of anti-TIM-3 antibodies. Any checkpoint intervention would also have to remain strictly adjunctive to timely antimicrobial therapy and source control, rather than delaying or replacing these established components of sepsis care [3].

Existing oncology antibodies should therefore be viewed as pharmacological starting points rather than ready-made sepsis therapeutics. None has been clinically validated or approved for sepsis. Repurposing would require indication-specific dose-ranging, PK/PD and receptor occupancy studies, clinically relevant delayed-treatment designs, prospective immune state enrichment, predefined stopping rules and careful surveillance of pathogen clearance and secondary infection. Because sepsis immune states can evolve faster than the biological effects of a long-acting antibody, the optimal modality may ultimately require shorter or more titratable target engagement; this remains an empirical question rather than an established design principle.

Future TIM-3 studies in sepsis should therefore be built around the paired interpretation of the receptor, ligand, cell type, immune phase, function and drug pharmacology. Without this integration, TIM-3 will remain easy to identify as an upregulated checkpoint but difficult to develop into a safe, pharmacologically controlled and clinically testable sepsis intervention.

## 10. Conclusions

Immune checkpoint biology in sepsis should be interpreted in relation to the level of evidence supporting each claim. Human studies have documented TIM-3 expression and associations with lymphoid dysfunction or apoptosis, while ex vivo blockade studies provide evidence that some immune defects can be functionally modified in isolated cells [15,64]. More direct causal evidence for TIM-3-associated NKT cell, hepatic CD8+ T-cell and CD4+ T-cell phenotypes is derived predominantly from CLP, receptor mutant or cell-specific knockout models [46,47,65]. In parallel, ligand-specific and myeloid TIM-3 mechanisms are informed partly by non-sepsis experimental systems [58,59,60,71,72]. These evidence levels are complementary but should not be treated as interchangeable, particularly when therapeutic implications are considered.

The emerging picture is therefore not that TIM-3 is uniformly detrimental or uniformly protective. Rather, its biological effect appears to depend on the checkpoint-expressing cell type, immune state, ligand context, tissue compartment and mode of experimental perturbation. This framework is consistent with broader sepsis endotyping studies showing that host response states can be measured and may modify responses to immunomodulatory therapy [26,30]. However, identifying an immune state does not establish that TIM-3 is the causal driver of this state, nor does it demonstrate that TIM-3 modulation will improve patient-centered outcomes.

For TIM-3, the immediate priority is therefore to identify which candidate circuits are reproducible in human sepsis, demonstrate causal and reversible dysfunction and establish pharmacological target engagement before clinical efficacy is inferred. Recent translational frameworks similarly emphasize predictive enrichment and the repeated assessment of immune status rather than one-size-fits-all immunomodulation [39]. On the basis of the current evidence, TIM-3 is best viewed as a candidate for mechanistically stratified investigation in sepsis rather than as an established therapeutic target.

## Figures and Tables

**Table 1:** Candidate operational framework for prospective TIM-3 circuit assignment in sepsis.

Domain/Candidate Marker	Assay and Sample	Proposed Use/Decision Rule	Feasibility	Key Limitations
Inflammatory burden Ferritin, IL-6, shock/SOFA	Routine chemistry or immunoassay; serum/plasma	Supports an inflammation-dominant state when innate and lymphoid function are relatively preserved [30].	Routine; usually same-day	Non-specific; affected by source, tissue injury and comorbidity. No TIM-3-specific cutoff.
Innate competence mHLA-DR; LPS-induced TNF	Quantitative flow ± ex vivo LPS stimulation; fresh whole blood	Low mHLA-DR with impaired inducible response supports an innate immunoparalytic component [19,28,30,74].	Same-day; specialized lab for functional assay	Requires standardized gating, calibration, stimulation conditions and processing time.
Lymphoid status ALC, subsets, apoptosis, IFN-γ/TNF	CBC + multiparameter flow ± ex vivo stimulation; whole blood/PBMCs	Persistent lymphopenia, apoptosis or impaired effector response supports a lymphoid dysfunction component [42,64].	CBC routine; extended flow/function same-day	Fresh samples and harmonized subset/stimulation protocols are required.
Cell-specific TIM-3	Multiparameter flow; whole blood/PBMCs	Not sufficient alone. A candidate circuit requires concordant cell-specific TIM-3, functional defect and plausible context [64].	Potentially same-day in specialized labs	Clone, gating, activation state, MFI vs. % positive and handling may alter results.
Ligand context Galectin-9, HMGB1, CEACAM1, phosphatidylserine	Protein immunoassay, flow or EV/tissue assays as appropriate	Exploratory supportive evidence; should not independently determine circuit assignment [58,59,60,61].	Mostly batch/research-grade	Compartment- and handling-sensitive; soluble and cell-associated forms are not equivalent; no validated sepsis cutoffs.
Suppressive myeloid compartment	Multiparameter flow; fresh whole blood/PBMCs	Persistent suppressive myeloid expansion with concurrent innate/lymphoid dysfunction supports a mixed suppressive state.	Same-day in specialized labs	No universally accepted sepsis MDSC-like gating definition.
Integrated assignment	Prespecified integration of longitudinal core + extended data	Dominant: concordant pattern with functional support. Mixed: ≥2 coexisting patterns without clear predominance. Indeterminate: discordant/incomplete evidence; no forced assignment.	Core panel for real-time decisions; extended panel may be batched	Rules must be prespecified and validated for reproducibility, temporal stability, functional concordance and incremental value.

Note: Feasibility categories are approximate and depend on local laboratory infrastructure. The proposed rules are for prospective validation and are not established clinical treatment criteria. Ligand assays should not currently delay enrollment or bedside decisions. Abbreviations: ALC, absolute lymphocyte count; CBC, complete blood count; EV, extracellular vesicle; IFN-γ, interferon-γ; LPS, lipopolysaccharide; MDSC, myeloid-derived suppressor cell; MFI, mean fluorescence intensity; mHLA-DR, monocytic human leukocyte antigen DR; PBMC, peripheral blood mononuclear cell; SOFA, Sequential Organ Failure Assessment; TIM-3, T-cell immunoglobulin and mucin-domain containing-3; TNF, tumor necrosis factor.

## Data Availability

No new data were created or analyzed in this study. Data sharing is not applicable to this article.

## Supplementary Materials

The following supporting information can be downloaded at https://www.mdpi.com/article/10.3390/pharmaceutics18101343/s1. Table S1: Literature search log for the narrative review.
