# Supplementary material for: Cell Type- and Phase-Dependent Immune Checkpoint Targeting in Sepsis: TIM-3 as a Model for Precision Immunomodulation
Source: Pharmaceutics. 2026 Oct 21;18(10):1343. doi: 10.3390/pharmaceutics18101343 (PMC13708479; doi:10.3390/pharmaceutics18101343)
Supplement: Supplementary file 1 [file pharmaceutics-18-01343-s001.zip › pharmaceutics-4575913-supplementary.pdf]

Supplementary Table S1. Literature search log for the narrative review

Database	Search date	Search strategy / concepts	Purpose
PubMed	2026-08-30	sepsis AND (TIM-3 OR HAVCR2)	Direct TIM-3–sepsis evidence
PubMed	2026-08-30	sepsis AND (immune checkpoint OR checkpoint blockade OR PD-1 OR PD-L1 OR CTLA-4 OR LAG-3 OR TIGIT OR BTLA)	Broader immune-checkpoint evidence in sepsis
PubMed	2026-08-30	sepsis AND (immune state OR immune endotype OR immunoparalysis OR immune dysfunction OR precision immunotherapy)	Immune-state, endotype, and precision-immunomodulation framework
PubMed	2026-08-30	TIM-3 AND (Galectin-9 OR HMGB1 OR CEACAM1 OR phosphatidylserine OR PtdSer)	TIM-3 ligand biology and microenvironmental context
PubMed	2026-08-30	TIM-3 AND (T cell OR CD4 OR CD8 OR NKT OR NK OR monocyte OR macrophage OR dendritic cell OR myeloid)	Cell type–specific TIM-3 biology
PubMed	2026-08-30	TIM-3 AND (clinical trial OR antibody OR blockade OR inhibitor OR pharmacology OR pharmacokinetics OR pharmacodynamics)	Translational pharmacology, intervention studies, and clinical development
Web of Science Core Collection	2026-08-30	sepsis AND (TIM-3 OR HAVCR2); citation tracking of key TIM-3 and sepsis studies	Cross-checking, citation expansion, and identification of related studies
Reference-list screening	Throughout review and revision	Reference lists of key primary studies, systematic reviews, and major mechanistic reviews were screened manually	Identification of additional relevant primary studies and mechanistic evidence not captured by keyword searches
